# Hormetic Concentrations of Hydrogen Peroxide but Not Ethanol Induce Cross-Adaptation to Different Stresses in Budding Yeast

**DOI:** 10.1155/2014/485792

**Published:** 2014-01-14

**Authors:** Halyna M. Semchyshyn

**Affiliations:** Department of Biochemistry and Biotechnology, Vassyl Stefanyk Precarpathian National University, 57 Shevchenko Street, Ivano-Frankivsk 76025, Ukraine

## Abstract

The biphasic-dose response of microorganisms to hydrogen peroxide is a phenomenon of particular interest in hormesis research. In different animal models, the dose-response curve for ethanol is also nonlinear showing an inhibitory effect at high doses but a stimulatory effect at low doses. In this study, we observed the hormetic-dose response to ethanol in budding yeast *S. cerevisiae*. Cross-protection is a phenomenon in which exposure to mild stress results in the acquisition of cellular resistance to lethal stress induced by different factors. Since both hydrogen peroxide and ethanol at low concentrations were found to stimulate yeast colony growth, we evaluated the role of one substance in cell cross-adaptation to the other substance as well as some weak organic acid preservatives. This study demonstrates that, unlike ethanol, hydrogen peroxide at hormetic concentrations causes cross-resistance of *S. cerevisiae* to different stresses. The regulatory protein Yap1 plays an important role in the hormetic effects by low concentrations of either hydrogen peroxide or ethanol, and it is involved in the yeast cross-adaptation by low sublethal doses of hydrogen peroxide.

## 1. Introduction

Organisms' adaptation to environmental stress has become a subject of great interest over the last decades [1–5]. Like other organisms, budding yeast *Saccharomyces cerevisiae* has developed several strategies to survive stressful changes in their environment. Sudden challenge can result in disturbance of cellular functions or even cell death. Clearly, yeast cells respond rapidly and modify their internal systems to prevent dramatic events. Depending on the intensity and type of stress, many different mechanisms contribute to the development of yeast resistance to stressful changes.

It is widely believed that cell exposure to mild stress results in the acquisition of cellular resistance to further lethal stress, what is called “adaptive response” or “preadaptation” [6, 7]. The phenomenon has been observed in various organisms: from bacteria to humans. In many cases, an exposure to mild stress develops tolerance not only to higher doses of the same stressor but also to stress caused by other factors. This phenomenon, known as “cross-protection” or “cross-adaptation” [6, 7], suggests the existence of complex mechanisms, which sense and respond to different kinds of stress. The literature includes data on *S. cerevisiae* general response, pre-adaptation, and cross-adaptation to extreme temperatures, osmotic shock, and oxidative stress [2, 6, 8–12].

There is information on the increased resistance to severe stress in yeast preexposed to mild sublethal stress, which requires the global-stress transcription factors Msn2/4p to regulate induction of the so-called environmental stress response genes [6, 11]. Activation of Msn2/4p, in particular, is an important way to induce antioxidant defense against hydrogen peroxide [13, 14]. Yap1p transcriptional regulatory protein also mediates an adaptive response of yeast to H_2_O_2_-induced stress [4, 9, 13–15]. Sublethal hormetic concentrations of hydrogen peroxide are believed to induce a protective response with increased resistance to subsequent lethal stress in yeast cells [6, 7, 10, 16]. However, unlike Msn2/4p, the potential role of Yap1p in the cross-adaptation phenomenon is poorly investigated.

Yap1p is found to be localized in the cytoplasm under nonstressful conditions, but upon exposure to hydrogen peroxide it rapidly translocates to the nucleus and changes the expression of target genes [17–19]. Although Yap1p was earlier suggested to play a minor role in the regulation of gene expression under ethanol stress [20], like H_2_O_2_, ethanol was recently found to trigger Yap1 nuclear accumulation and activate some antioxidant enzymes in *S. cerevisiae* [21].

In the present study, we have shown that low hormetic concentrations of hydrogen peroxide as well as ethanol stimulated yeast colony growth. Therefore, we evaluated the role of one substance in cell cross-adaptation to the other substance as well as some weak organic acid preservatives, and potential role of Yap1p in yeast cross-adaptation by hormetic concentrations of hydrogen peroxide and ethanol to stressful conditions.

## 2. Materials and Methods

### 2.1. Yeast Strains and Growth Conditions

The *Saccharomyces cerevisiae* strains used in this study are YPH250 (wild type: *MAT* **a** *trp1-Δ1 his3-Δ200 lys2-801 leu2-Δ1 ade2-101 ura3-52*) and its isogenic derivative ΔYAP1 (YPH250 *yap1Δ::HIS3*) described earlier [22]. The strains were kindly provided by Professor Yoshiharu Inoue (Kyoto University, Japan).

Yeast cells were grown in Erlenmeyer flasks containing YPD liquid medium (1% yeast extract, 2% peptone, and 2% glucose) in a volume that respected the ratio 1 : 5 regarding media volume to flask volume. Cells were grown with shaking at 175 r.p.m., 28°C, and pH 7.0 for 24 h to late exponential phase (*A*_600_ ~ 0.8-0.9).

### 2.2. Preincubation and Stress Induction

The experimental culture after growth under the conditions mentioned above was split into three groups: one exposed to stress, another preadapted by low concentrations of hydrogen peroxide or ethanol and then exposed to stress, and the last one serving as a control to which none of the abovementioned substances was added.

For stress induction, aliquots of experimental culture were incubated at 28°C with 50 mM H_2_O_2_ for 30 min, 15% or 20% ethanol for 60 min, and 200 mM acetic or 100 mM propionic acid for 120 min. At low pH, acetic acid (p*K*_a_ 4.75) and propionic acid (p*K*_a_ 4.88) exist mainly in the undissociated state, in which they enter the cell rather easily [23]. For acid stress, the pH value of YPD medium was adjusted to 3.0 with HCl in order to reach maximum penetration of the acids into cells [24, 25]. Under stress induced by hydrogen peroxide or ethanol the pH value of YPD medium was 7.0.

To study the preadaptation effect on cell survival under stress, aliquots of experimental culture were preincubated with 0.05, 0.25, and 0.5 mM H_2_O_2_ or 1%, 2.5%, and 5% ethanol at 28°C and pH 7.0 for 30 or 60 minutes, respectively.

Control cells were incubated under the same conditions but without hydrogen peroxide, ethanol, acetate, or propionate. In preliminary experiments, it was shown that yeast colony growth was virtually the same at pH 3.0 and 7.0 [7, 26]. Thus, control cells for acetate- and propionate-induced stress were incubated in YPD medium at pH 3.0 without organic acids.

### 2.3. Evaluation of Yeast Colony Growth and Statistical Analysis

Yeast colony growth was analyzed by plating in triplicate on YPD agar after proper dilution. The plates were incubated at 28°C for 3 days and the colony forming units (CFU) were counted [27]. Yeast colony growth was expressed as percentage of total amount of respective control cells plating on YPD agar.

Experimental data are expressed as the mean value of 4–6 independent experiments ± the standard error of the mean (SEM), and statistical testing used Student's *t*-test.

## 3. Results and Discussion

**Figure 1:**
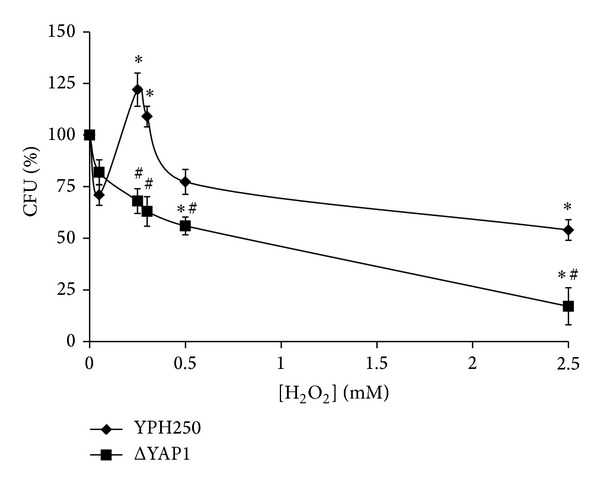
Colony forming units of *S. cerevisiae* YPH250 and its derivative ΔYAP1 after exposure to different concentrations of hydrogen peroxide. Significantly different from respective values obtained for *S. cerevisiae* YPH250 with *P* < 0.05^#^, and for corresponding cells treated with 0.05 mM H_2_O_2_ with *P* < 0.05*. Data are mean ± SEM (*n* = 4–6).

The biphasic-dose response to hydrogen peroxide is the phenomenon of particular interest in hormesis research [8, 28–31]. In the case of budding yeast, special attention is focused on the concentrations of hydrogen peroxide (≤0.4 mM) found to stimulate yeast colony growth by about 30% (stimulatory/hormetic zone) [8]. At the same time, hydrogen peroxide has been shown to induce cell toxicity at concentrations higher than 0.5 mM. Earlier, the nonlinear dependence of *S. cerevisiae* RZ53-6 survival on different concentrations of hydrogen peroxide was demonstrated by Davies and colleagues [8]. In the present study, we observed nonlinear dependence of *S. cerevisiae* YPH250 viability on different levels of H_2_O_2_ (Figure 1). Since H_2_O_2_ low concentrations (≤0.5 mM) inhibited significantly the colony growth of *Δyap1* mutant derived from YPH250 wild type (Figure 1), we suggested Yap1p involvement in hormetic effect by low doses of hydrogen peroxide in the parental strain.

**Figure 2:**
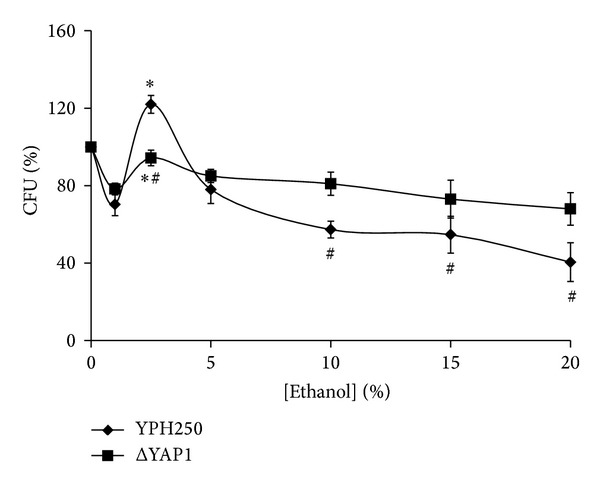
Colony forming units of *S. cerevisiae* YPH250 and its derivative ΔYAP1 after exposure to different concentrations of ethanol. Significantly different from respective values obtained for *S. cerevisiae* YPH250 with *P* < 0.05^#^, and for corresponding cells treated with 1% ethanol with *P* < 0.05*. Data are mean ± SEM (*n* = 4–6).

In different animal models, the dose-response curve for ethanol is also biphasic showing an inhibitory effect at high doses but a stimulatory effect at low doses [32, 33]. We observed non-linear dose response to ethanol in *S. cerevisiae* YPH250 wild strain (Figure 2). Yeast ability to form colonies in cultures treated with 1% ethanol was decreased by 30% comparing to untreated control cells. Cells exposed to 2.5% ethanol demonstrated percent colony growth that exceeded the original control value by about 20%. At the higher concentrations used (≥5%), ethanol significantly inhibited yeast colony growth.

Figure 2 demonstrates also the influence of different concentrations of ethanol on the reproductive ability of *Δyap1* mutant isogenic derivative of YPH250. Generally, the effect is somewhat similar to that obtained for YPH250 parental strain. Exposure of *Δyap1* cells to 1% ethanol decreased colony growth by 22%. It should be noted that 2.5% ethanol increased the parameter comparing with that found for cells exposed to 1% ethanol, but colony growth of the *Δyap1* cells treated with 2.5% ethanol did not exceed the initial control value. We also supposed that Yap1 regulatory protein was involved in some way in the stimulation of yeast colony growth under stress induced by 2.5% ethanol (Figure 2).

The Yap1 transcription factor controls the expression of over 150 genes in the response of *S. cerevisiae* to hydrogen peroxide [34–36]. Since most of them were found to play an important role in yeast survival under H_2_O_2_-induced stress, we expected that Yap1p was involved in hormetic effect by hydrogen peroxide. At the same time, there are somewhat controversial data regarding Yap1p activation by ethanol. Earlier Yap1p was suggested to play a minor role in regulation of gene expression under ethanol stress in *S. cerevisiae* [37]; however, ethanol was recently found to trigger Yap1 nuclear accumulation and to activate some antioxidant enzymes in the yeast [21].

**Figure 3:**
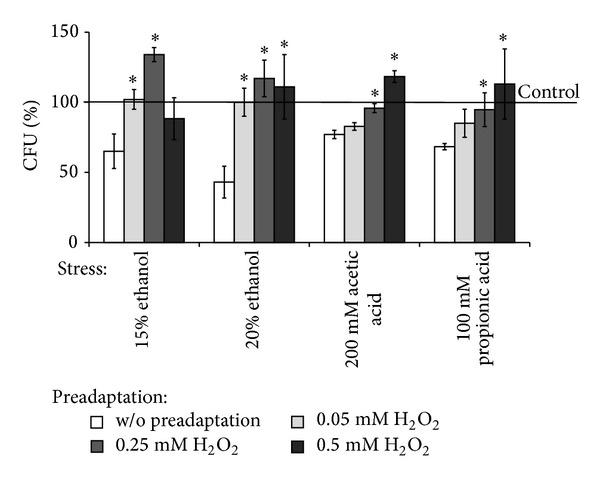
Colony forming units of *S. cerevisiae* YPH250 cells pretreated with low concentrations of  H_2_O_2_ under exposure to different stresses. *Significantly different from respective values obtained for cells with *P* < 0.05. Data are mean ± SEM (*n* = 5-6).

Since both hydrogen peroxide and ethanol at low concentrations were found to stimulate the colony growth of* S. cerevisiae* wild type, next we evaluated the role of one substance in cell cross-adaptation to the other substance as well as some weak organic acid preservatives.  Figure 3 shows the effect of yeast pretreatment with low H_2_O_2_ concentrations on its reproductive ability under stress induced by ethanol, acetate, and propionate. As seen in the figure, all the stressors significantly inhibited colony growth of *S. cerevisiae* YPH250 (by 35%, 57%, 23%, and 32% in response to 15% or 20% ethanol and 200 mM acetate or 100 mM propionate, resp.). Yeast pretreatment with hormetic doses of hydrogen peroxide markedly increased reproductive ability under the stressful conditions. For example, the highest colony growth (134% and 118% comparing to control untreated cells) was observed in yeast preincubation with 0.25 mM H_2_O_2_ and then stressed by 15% or 20% ethanol. In the case of weak organic acids, the highest reproductive ability was observed in yeast pre-treated with 0.5 mM H_2_O_2_ (118% and 113% for acetate- and propionate-exposed cells, resp.).

Hydrogen peroxide is known to affect expression of a variety of genes involved in signal transduction, transcriptional regulation, antioxidant defence, and protein, carbohydrates, or lipid metabolism in different organisms [9, 12–14, 29, 35–39]. That is why at low hormetic concentrations, H_2_O_2_ may act as an important signal molecule stimulating various defensive mechanisms and enhancing survival of yeast cells under lethal stress.

**Figure 4:**
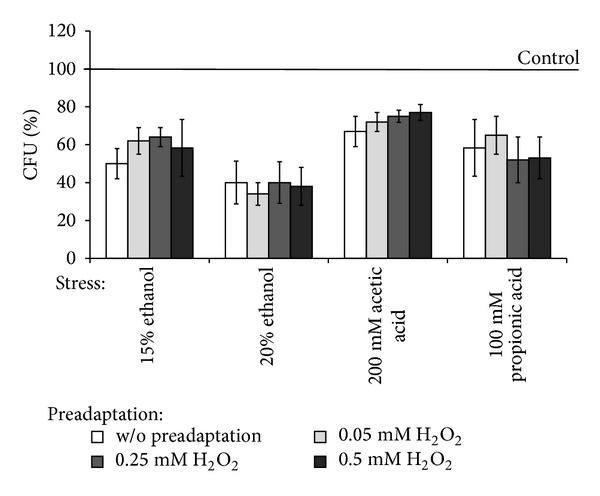
Colony forming units of ΔYAP1 mutant pretreated with low concentrations of H_2_O_2_ under exposure to different stresses. Data are mean ± SEM (*n* = 4–6).

It should be noted that, comparing to the wild type, *Δyap1* mutant demonstrated significantly higher sensitivity to all the types of stressors and no cross-adaptation by low concentrations of hydrogen peroxide to any stressors used (Figure 4). The latter confirms an important role of Yap1p in cross-adaptation effect by hormetic doses of hydrogen peroxide in parental YPH250 strain.

**Figure 5:**
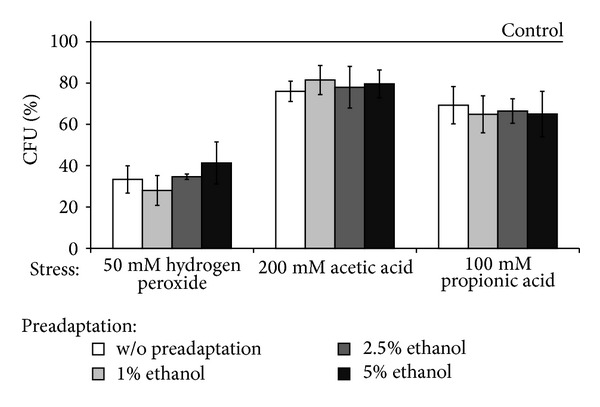
Colony forming units of *S. cerevisiae* YPH250 cells pretreated with low concentrations of ethanol under exposure to different stresses. Data are mean ± SEM (*n* = 5-6).

**Figure 6:**
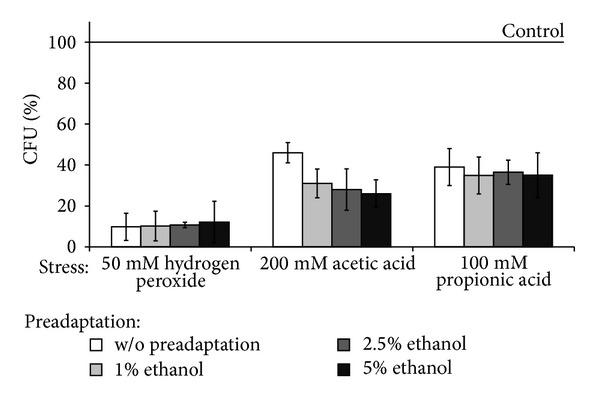
Colony forming units of ΔYAP1 mutant pretreated with low concentrations of ethanol under exposure to different stresses. Data are mean ± SEM (*n* = 4-5).

Earlier it was suggested that depending on strain genotype, ethanol either provided or did not provide yeast protection against environmental stress [6]. Despite the fact that yeast cells demonstrated non-linear dose response to ethanol (Figure 2), we did not observe any stimulatory effect by ethanol preadaptation on colony growth of yeast under stressful conditions (Figure 5). As seen, 50 mM H_2_O_2_, 200 mM acetate, and 100 mM propionate inhibited *S. cerevisiae* YPH250 ability to form colonies by 33%, 76%, and 69%, respectively, and preexposure of yeast by ethanol did not change the parameter. As seen in Figure 6, *Δyap1* mutant demonstrated much higher sensitivity to all the types of stressors comparing to wild type as well as no cross-adaptation by ethanol to any stressors used.

## 4. Conclusion

This study demonstrates that, unlike ethanol, hormetic concentrations of hydrogen peroxide cause cross-resistance of *S. cerevisiae* YPH250 to different stresses. The regulatory protein Yap1 plays an important role in the hormetic effects caused by low concentrations of either hydrogen peroxide or ethanol, and it is involved in the yeast cross-adaptation to stressful conditions by low sublethal doses of hydrogen peroxide.
